# Economic Burden of Disease-Associated Malnutrition at the State Level

**DOI:** 10.1371/journal.pone.0161833

**Published:** 2016-09-21

**Authors:** Scott Goates, Kristy Du, Carol A. Braunschweig, Mary Beth Arensberg

**Affiliations:** 1 Abbott Nutrition, Research & Development, Columbus, Ohio, United States of America; 2 Division of Nutritional Sciences, University of Illinois at Urbana-Champaign, Champaign, Illinois, United States of America; 3 Abbott Nutrition, Research Park at the University of Illinois, Champaign, Illinois, United States of America; 4 Department of Kinesiology and Nutrition, University of Illinois at Chicago, Chicago, Illinois, United States of America; 5 Abbott Nutrition Products Division of Abbott, Columbus, Ohio, United States of America; Johns Hopkins University Bloomberg School of Public Health, UNITED STATES

## Abstract

**Background:**

Disease-associated malnutrition has been identified as a prevalent condition, particularly for the elderly, which has often been overlooked in the U.S. healthcare system. The state-level burden of community-based disease-associated malnutrition is unknown and there have been limited efforts by state policy makers to identify, quantify, and address malnutrition. The objective of this study was to examine and quantify the state-level economic burden of disease-associated malnutrition.

**Methods:**

Direct medical costs of disease-associated malnutrition were calculated for 8 diseases: Stroke, Chronic Obstructive Pulmonary Disease, Coronary Heart Failure, Breast Cancer, Dementia, Musculoskeletal Disorders, Depression, and Colorectal Cancer. National disease and malnutrition prevalence rates were estimated for subgroups defined by age, race, and sex using the National Health and Nutrition Examination Survey and the National Health Interview Survey. State prevalence of disease-associated malnutrition was estimated by combining national prevalence estimates with states’ demographic data from the U.S. Census. Direct medical cost for each state was estimated as the increased expenditures incurred as a result of malnutrition.

**Principal Findings:**

Direct medical costs attributable to disease-associated malnutrition vary among states from an annual cost of $36 per capita in Utah to $65 per capita in Washington, D.C. Nationally the annual cost of disease-associated malnutrition is over $15.5 billion. The elderly bear a disproportionate share of this cost on both the state and national level.

**Conclusions:**

Additional action is needed to reduce the economic impact of disease-associated malnutrition, particularly at the state level. Nutrition may be a cost-effective way to help address high health care costs.

## Introduction

Disease-associated malnutrition (DAM) is malnutrition that occurs from disease-related causes. This is different from malnutrition caused by lack of availability of food. In patients with DAM, nutrient intake is diminished and inflammatory responses increase [1], inducing increased metabolic demand, decreased appetite, gastrointestinal problems, and difficulty chewing and swallowing, all of which can decrease lean body mass and increase the risks of complications during treatment of the primary disease [2]. Increased inflammatory responses also diminish immune response, increasing infection rates, decreasing muscle strength, retarding wound healing, and reducing physical function [3]. Collectively these factors increase risks for functional disability, frailty, and falling [4].

Related to malnutrition, sarcopenia (age-associated decrease in muscle mass and function) is commonly described as a concern for the institutionalized elderly. Emerging evidence suggests it is also a concern among free-living elderly [5–7]. Sarcopenic obesity, characterized by low lean body mass in obese individuals, is particularly under-recognized as the excess fat deposits hide the wasted lean body mass in “plain sight.” Among ICU patients, prevalence of sarcopenia has been documented at 56–71%, with 46% or more categorized as overweight/obese by BMI [8,9]. Approximately 30% of liver failure patients with sarcopenia are overweight/obese [10].

Malnutrition is a largely under-recognized health problem. Greater than one-third of patients are malnourished prior to being admitted to the hospital [11]. Further, one-third of patients not malnourished at the time of admission, become malnourished during their stay at the hospital [12]. While DAM impacts patient functionality and health outcomes, it is not just a problem for patients and their families. Because of the economic burden it places on the healthcare system, DAM is also an important concern for society, especially for healthcare providers and policy makers. DAM increases the overall costs of care, increasing complications, extending hospital stays, and elevating rates of readmissions. The American Society for Parenteral and Enteral Nutrition (A.S.P.E.N.) recently proposed that “addressing disease-[associated] malnutrition in hospitalized patients should be a national goal in the United States…to improve patient outcomes by reducing morbidity, mortality, and costs… [and] to alert health care organizations on the need to provide optimal nutrition care.” Further, they noted “Nutrition intervention has been shown to improve clinical outcomes in many studies, most often in patients 65 years of age or older who are malnourished or at risk for developing malnutrition.” [13].

Health economic studies are important for investigating the role that malnutrition care can play in reducing costs. Health economics research has traditionally been focused on medical treatments and therapies, however the number of economic studies of nutrition and malnutrition is expanding [14]. Several studies have estimated the direct medical cost of DAM on the international or national level. Most notably, a study by Inotai et al. estimated in 2009 the direct medical cost burden of DAM in Europe was over €31 billion [15]. A similar study by Snider et al. estimated DAM in the United States had an annual burden of $9.5 billion in direct medical cost (2010 dollars) [16].

These national and international estimates of spending are valuable in understanding the magnitude of DAM on a national scale, however, many policy actions to address malnutrition take place at the local and/or state levels. To make informed decisions, state policy makers must understand the cost of DAM in their jurisdiction. The objective of our study is to examine and quantify state-level economic burden (measured in direct medical costs) of disease-associated malnutrition in the United States, to help policy makers more completely understand the magnitude of the problem and provide support for policy changes needed to better identify, prevent, and treat malnutrition.

## Methods

We estimate state-level direct medical costs of DAM for 8 diseases that were previously included in the economic studies by Inotai et al. and Snider et al.: breast cancer, chronic obstructive pulmonary disorder (COPD), colorectal cancer (CRC), coronary heart disease (CHD), dementia, depression, musculoskeletal disorders (MSD) and stroke. Direct medical costs were estimated using similar methodology as Inotai et al. [15] and Snider et al. [16], with necessary adjustments to enable results at the state level. High prevalence diseases were deliberately avoided because, as noted by Snider et al., “malnourished individuals may have more than 1 disease” therefore “counting the burden of DAM across several high prevalence diseases (…) would likely lead to counting some of the same malnourished individuals more than once.”[16].

Consistent with Snider et al. [16] and Somanchi et al. [17], malnutrition was defined as having less than 90% of ideal body weight [18], and/or serum albumin levels less than 3.5 g/dL [19]. Ideal body weight was identified using the traditional Hamwi [20] equation: 106 lbs + 6 lbs/inch over 5 feet for men; 100 lbs + 5 lbs/inch over 5 feet for women. Low albumin has been shown to predict mortality, but it can be affected by factors other than nutritional status, including inflammation [21]. However, concerns for the profound fluctuations in albumin that accompany acute illness are somewhat diminished because the National Health and Nutrition Examination Survey (NHANES) used for our analysis excludes institutionalized participants. Further, inclusion of albumin in our economic model enables comparability between our findings and previous studies in this area. All other disease definitions used in our study are based on those used by Snider et al. [16] and are listed in S1 Appendix.

State-level direct medical cost of DAM was calculated in five steps. First, the prevalence of malnutrition (as previously defined to be less than 90% of ideal body weight and/or serum albumin levels less than 3.5 g/dL) within each of the 8 diseases was calculated using data from the NHANES 2009–2014 [22]. Different prevalence rates were estimated for 30 groups defined by age (< = 18, 19–45, 46–55, 56–64, > = 65), sex (male, female) and race (white, black, other).

Second, disease prevalence for each age-sex-race group was calculated. We used the National Health Interview Survey (NHIS) when possible because of its larger sample size [23]. When disease definitions from NHIS did not closely match the definition in NHANES, or could not be found, NHANES data was used to estimate disease prevalence (all child disease prevalence and adult dementia and depression rates were estimated with NHANES). NHIS definitions for all disease rates can be found in S1 Appendix and are based on those used by Snider et al. [16].

Third, state population estimates for each age-sex-race group were obtained from the U.S. Census [24].

Fourth, estimates of the average direct medical cost for each condition, and the proportional increase attributable to malnutrition were identified from the literature. These estimates are provided in S1 Appendix.

Finally, the total state-level direct medical cost of DAM for each condition was estimated using the following equation:

$$Total\;Cost\;of\;DAM=\underset{i}{\sum }\underset{j}{\sum }C_{i}*\left(\frac{PMN_{ij}*\Delta MN}{1+PMN_{ij}*\Delta MN}\right)*\rho_{ij}*POP_{j}$$

$$\begin{array}{l} Where:\\ C_{i}\;is\;the\;average\;direct\;medical\;spending\;of\;disease\;i\\ PMN_{ij}is\;the\;prevalence\;of\;malnutrition\;in\;disease\;i\;for\;group\;j\\ \Delta MN\;is\;the\;ratio\;of\;costs\;between\;malnurioushed\;individuals\;and\;non-malnourished\;individuals\\ \rho_{ij}\;is\;the\;prevalence\;of\;disease\;i\;in\;group\;j\\ POP_{j}\;is\;the\;number\;of\;individuals\;in\;group\;j \end{array}$$

### Sensitivity Analysis

A probabilistic sensitivity analysis was conducted using Monte Carlo simulation. Both the NHIS and NHANES surveys were probabilistically recreated using the -svybsamp2- program in Stata 13.1 [25] to resample the data and preserve the same survey structure of the original surveys [26]. Cost parameters were randomly drawn from a gamma distribution when adequate information was reported to allow specification. When such information was not available, costs were drawn from a uniform distribution with the upper and lower bounds 20% away from the mean. The simulation was repeated 1000 times and results are reported as 90% confidence intervals. Details on parameters used in the sensitivity analysis can be found in S1 Appendix.

## Results

Estimates of the state level burden of direct medical spending on DAM are presented in Table 1. California has the largest burden of DAM with direct medical expenditures of over $1.7 billion annually. Texas, Florida and New York also face a significant burden of DAM with expenditures of over $1 billion annually.

**Table 1:** Estimated Direct Medical Cost of Disease-Associated Malnutrition.

State	Results (90% Confidence Interval)	Per Capita Cost	Results (65+)	Per Capita Cost (65+)
**Alabama**	$267,015,920 ($218,238,816, $323,718,080)	$54 ($44, $66)	$71,968,488 ($62,916,296, $81,849,280)	$96 ($84, $109)
**Alaska**	$32,631,558 ($25,892,390, $39,326,528)	$41 ($33, $50)	$7,091,582 ($6,163,682, $8,092,297)	$100 ($87, $114)
**Arizona**	$303,535,808 ($241,320,896, $370,999,104)	$44 ($35, $54)	$95,796,376 ($84,122,576, $108,166,688)	$89 ($78, $100)
**Arkansas**	$146,998,480 ($118,559,656, $179,101,792)	$49 ($39, $59)	$42,740,348 ($37,592,736, $48,380,896)	$91 ($80, $103)
**California**	$1,779,335,552 ($1,434,078,336, $1,434,078,336	$44 ($36, $53)	$492,571,488 ($428,707,904, $560,523,904)	$97 ($85, $111)
**Colorado**	$236,723,888 ($185,570,432, $291,377,216)	$43 ($34, $53)	$60,418,264 ($53,125,408, $68,220,496)	$88 ($77, $99)
**Connecticut**	$177,031,824 ($142,032,288, $215,684,848)	$48 ($39, $59)	$49,822,676 ($43,874,712, $56,343,884)	$89 ($78, $101)
**Delaware**	$50,753,544 ($41,130,608, $61,685,976)	$53 ($43, $64)	$14,656,278 ($12,840,961, $16,652,654)	$95 ($83, $107)
**District of Columbia**	$44,293,000 ($35,309,632, $53,729,096)	$65 ($52, $79)	$9,390,157 ($8,006,677, $10,949,040)	$124 ($106, $144)
**Florida**	$1,061,692,992 ($859,051,456, $1,287,815,168)	$52 ($42, $63)	$346,982,176 ($305,121,152, $392,876,000)	$91 ($80, $103)
**Georgia**	$549,650,240 ($444,891,712, $665,635,456)	$53 ($43, $65)	$125,373,000 ($109,428,864, $143,051,600)	$99 ($87, $113)
**Hawaii**	$84,797,592 ($66,575,184, $103,866,368)	$45 ($35, $55)	$31,726,716 ($26,612,724, $37,030,492)	$124 ($104, $145)
**Idaho**	$67,714,216 ($52,564,512, $83,998,560)	$40 ($31, $50)	$20,377,088 ($17,931,348, $22,986,322)	$87 ($76, $98)
**Illinois**	$630,516,352 ($509,110,272, $768,060,928)	$48 ($39, $58)	$167,950,480 ($147,227,424, $190,724,032)	$93 ($82, $106)
**Indiana**	$304,094,912 ($242,018,656, $373,562,528)	$45 ($36, $56)	$83,279,520 ($73,321,136, $94,107,792)	$88 ($77, $99)
**Iowa**	$137,240,256 ($107,786,048, $169,422,864)	$43 ($34, $54)	$41,757,180 ($36,806,488, $47,026,708)	$85 ($75, $95)
**Kansas**	$129,648,296 ($102,516,616, $159,097,952)	$43 ($34, $53)	$36,615,524 ($32,236,882, $41,342,440)	$87 ($77, $99)
**Kentucky**	$205,388,224 ($163,026,080, $252,522,880)	$46 ($36, $56)	$57,608,312 ($50,715,280, $65,077,224)	$87 ($77, $99)
**Louisiana**	$261,012,528 ($212,181,280, $316,236,928)	$55 ($45, $67)	$63,654,108 ($55,524,952, $72,593,456)	$100 ($87, $114)
**Maine**	$62,515,084 ($48,874,552, $77,913,512)	$46 ($36, $58)	$20,817,142 ($18,348,924, $23,451,438)	$85 ($75, $96)
**Maryland**	$340,440,992 ($277,990,464, $409,653,696)	$55 ($45, $67)	$84,344,672 ($73,401,728, $96,475,184)	$102 ($88, $116)
**Massachusetts**	$322,609,120 ($258,172,224, $395,243,136)	$47 ($37, $57)	$90,326,104 ($79,511,424, $102,069,488)	$88 ($78, $100)
**Michigan**	$497,511,168 ($400,754,560, $606,731,904)	$49 ($39, $60)	$141,127,008 ($124,048,904, $159,935,344)	$92 ($80, $104)
**Minnesota**	$245,311,456 ($194,417,696, $301,950,656)	$44 ($35, $54)	$67,790,480 ($59,669,016, $76,483,360)	$87 ($76, $98)
**Mississippi**	$173,332,464 ($140,200,848, $210,837,024)	$57 ($46, $70)	$43,148,340 ($37,627,920, $49,206,216)	$100 ($87, $114)
**Missouri**	$293,064,128 ($235,087,856, $358,443,904)	$47 ($38, $58)	$84,043,568 ($74,010,832, $95,064,560)	$89 ($79, $101)
**Montana**	$46,296,536 ($36,322,560, $57,078,144)	$44 ($35, $54)	$15,137,121 ($13,304,658, $17,096,334)	$88 ($77, $99)
**Nebraska**	$82,157,576 ($64,721,576, $100,969,312)	$43 ($34, $53)	$23,409,542 ($20,614,788, $26,391,860)	$86 ($76, $97)
**Nevada**	$135,586,784 ($109,772,848, $164,513,984)	$46 ($37, $55)	$38,848,784 ($33,960,664, $44,128,448)	$95 ($83, $108)
**New Hampshire**	$60,858,696 ($47,326,696, $75,956,088)	$45 ($35, $56)	$18,116,064 ($15,962,738, $20,413,906)	$85 ($75, $96)
**New Jersey**	$448,747,296 ($364,723,456, $542,632,384)	$49 ($40, $59)	$124,254,664 ($108,837,232, $141,066,176)	$94 ($82, $107)
**New York**	$1,025,842,688 ($833,381,888, $1,238,834,688)	$51 ($41, $61)	$28,918,304 ($25,400,244, $32,649,880)	$90 ($79, $101)
**New Mexico**	$92,487,560 ($73,218,128, $112,826,432)	$43 ($34, $53)	$281,050,912 ($245,926,528, $319,588,416)	$96 ($84, $109)
**North Carolina**	$525,503,904 ($426,140,608, $638,225,536)	$52 ($42, $63)	$140,348,592 ($122,879,288, $159,536,720)	$95 ($83, $108)
**North Dakota**	$32,036,090 ($24,984,198, $39,484,780)	$42 ($33, $52)	$9,025,682 ($7,951,615, $10,175,980)	$86 ($75, $97)
**Ohio**	$568,419,008 ($456,125,568, $695,443,200)	$48 ($38, $59)	$162,532,560 ($143,131,392, $183,908,032)	$90 ($79, $102)
**Oklahoma**	$183,027,776 ($147,502,800, $221,717,952)	$44 ($36, $54)	$53,003,912 ($46,518,312, $60,020,880)	$92 ($81, $104)
**Oregon**	$181,030,208 ($142,299,376, $222,707,280)	$44 ($34, $54)	$56,126,272 ($49,330,920, $63,368,580)	$87 ($77, $99)
**Pennsylvania**	$636,048,768 ($512,372,960, $777,165,632)	$49 ($39, $60)	$190,557,488 ($167,795,824, $215,459,136)	$89 ($78, $100)
**Rhode Island**	$50,781,376 ($40,460,728, $62,291,192)	$47 ($37, $57)	$14,485,806 ($12,753,338, $16,341,575)	$87 ($76, $98)
**South Carolina**	$271,378,304 ($221,157,280, $328,374,400)	$55 ($45, $67)	$74,782,944 ($65,337,404, $85,138,232)	$98 ($85, $111)
**South Dakota**	$37,129,324 ($29,218,250, $45,537,900)	$43 ($33, $52)	$11,368,387 ($10,000,352, $12,835,862)	$87 ($76, $98)
**Tennessee**	$330,590,784 ($266,203,136, $403,762,624)	$50 ($40, $61)	$90,469,296 ($79,539,880, $102,467,152)	$91 ($80, $103)
**Texas**	$1,212,168,064 ($975,931,200, $1,480,503,552)	$44 ($36, $54)	$287,602,336 ($252,581,472, $325,920,928)	$92 ($81, $104)
**Utah**	$108,943,024 ($84,096,976, $135,957,696)	$36 ($28, $45)	$25,761,394 ($22,665,116, $29,081,360)	$87 ($76, $98)
**Vermont**	$29,008,612 ($22,573,144, $36,129,312)	$45 ($35, $57)	$9,114,263 ($8,031,334, $10,268,387)	$85 ($75, $96)
**Virginia**	$434,973,696 ($352,716,416, $527,751,872)	$51 ($41, $62)	$111,438,624 ($97,540,704, $126,780,296)	$96 ($84, $110)
**Washington**	$323,034,816 ($256,845,472, $393,669,248)	$44 ($35, $53)	$90,820,496 ($79,796,952, $102,606,704)	$90 ($79, $102)
**West Virginia**	$87,293,488 ($68,884,136, $107,860,592)	$46 ($37, $57)	$28,455,174 ($25,064,588, $32,076,444)	$86 ($76, $97)
**Wisconsin**	$265,044,816 ($210,049,024, $326,058,368)	$45 ($36, $56)	$76,195,096 ($67,076,304, $85,966,336)	$87 ($76, $98)
**Wyoming**	$25,271,312 ($19,628,788, $31,385,668)	$42 ($33, $53)	$7,177,984 ($6,313,404, $8,100,528)	$87 ($77, $98)
**National**	$15,598,520,320 ($12,632,376,320, $18,970,537,984)	$48 ($39, $58)	$4,320,378,880 ($3,790,066,688, $4,900,164,608)	$93 ($81, $105)

**Fig 1:**
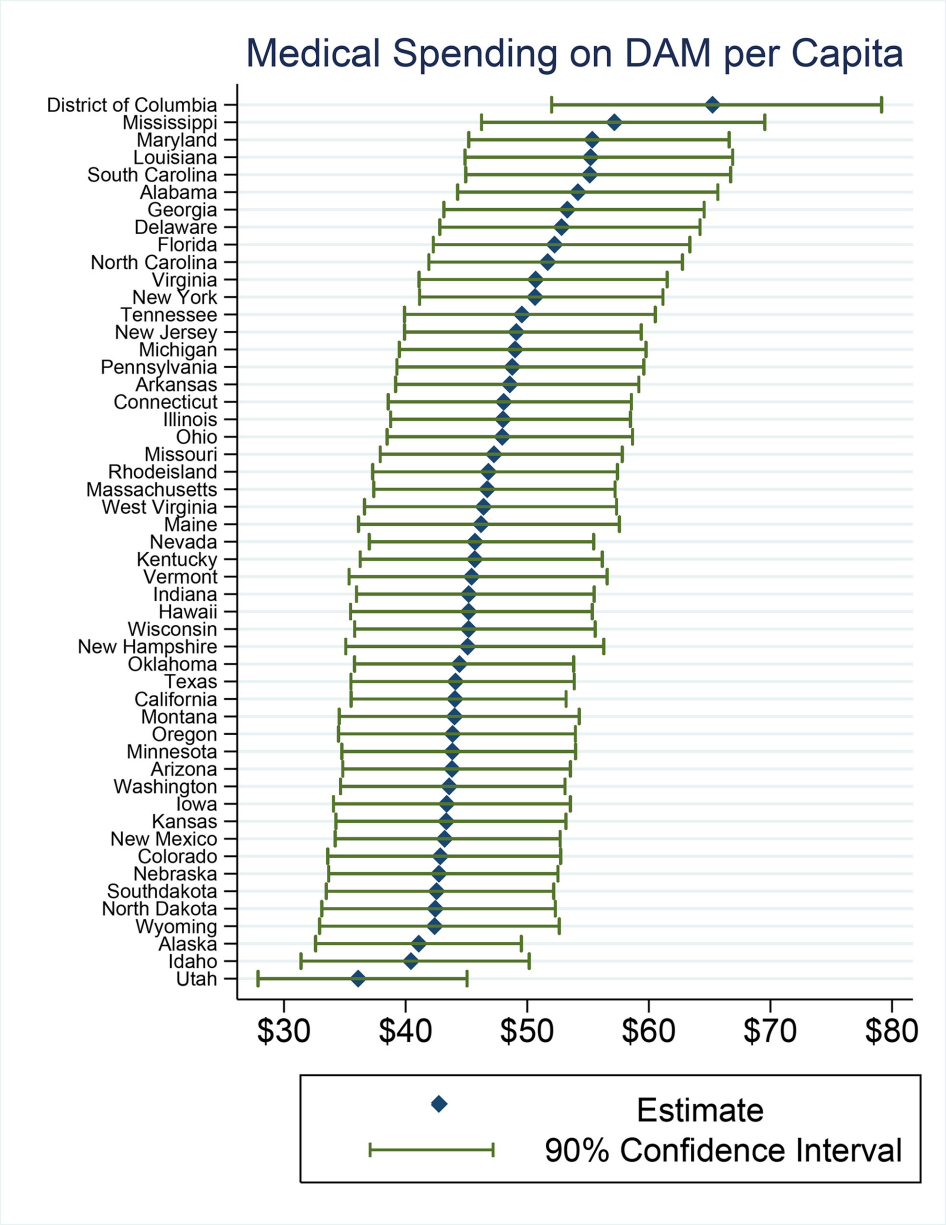
Medical Spending on DAM per Capita.

It is not surprising that larger states, such as those listed above, would face the highest burden of DAM. A more relevant statistic may be direct medical expenditures per capita attributable to DAM (Fig 1). Our analysis shows significant variation in state level spending per capita. Utah has the lowest burden at $36 per capita and Washington D.C. the highest with $65 per capita.

While the focus of this paper has been to estimate the state level burden of DAM, it is useful to look at national results as well, both to compare our results to previous studies and to better understand which diseases are the most costly contributors of DAM (Table 2).

**Table 2:** Burden of Direct Medical Expenditures related to Malnutrition by Disease (Million Dollars). Monte Carlo Simulation Confidence Intervals (90%) in brackets.^1^

State	Stroke	COPD	CHF	Colon Cancer	Breast Cancer	Dementia	Musculo-skelatal	Depression	Total
**Alabama**	$22.0 ($14.1, $30.3)	$30.7 ($22.5, $39.8)	$8.7 ($6.0, $12.1)	$3.0 ($1.2, $5.4)	$1.1 ($0.2, $2.1)	$152.4 ($111.0, $202.0)	$10.6 ($4.6, $17.3)	$38.4 ($26.7, $52.9)	$267.0 ($218.2, $323.7)
**Alaska**	$1.9 ($1.1, $2.8)	$3.0 ($2.2, $4.0)	$1.0 ($0.7, $1.3)	$0.3 ($0.1, $0.6)	$0.1 ($0.0, $0.2)	$17.8 ($12.8, $23.6)	$1.8 ($0.8, $2.9)	$6.7 ($4.4, $9.5)	$32.6 ($25.9, $39.3)
**Arizona**	$21.8 ($12.6, $30.7)	$33.8 ($24.6, $44.5)	$11.8 ($8.0, $16.6)	$4.1 ($1.3, $7.4)	$1.8 ($0.2, $3.4)	$165.2 ($118.1, $221.9)	$15.3 ($6.6, $25.4)	$49.7 ($33.7, $69.6)	$303.5 ($241.3, $371.0)
**Arkansas**	$11.5 ($7.2, $16.0)	$16.8 ($12.5, $21.6)	$5.3 ($3.7, $7.4)	$1.8 ($0.6, $3.3)	$0.8 ($0.1, $1.4)	$82.1 ($59.5, $109.7)	$6.5 ($2.7, $10.9)	$22.1 ($15.2, $31.0)	$147.0 ($118.6, $179.1)
**California**	$120.8 ($72.9, $169.7)	$184.7 ($137.7, $238.1)	$56.7 ($40.1, $78.7)	$21.5 ($7.1, $39.5)	$8.6 ($2.4, $15.2)	$988.6 ($717.9, $1,304.2)	$91.3 ($41.2, $147.7)	$307.0 ($210.8, $426.2)	$1,779.3 ($1,434.1, $2,148.1)
**Colorado**	$16.3 ($9.2, $23.4)	$25.0 ($18.0, $33.0)	$8.2 ($5.7, $11.5)	$2.6 ($0.8, $4.7)	$1.1 ($0.1, $2.2)	$130.6 ($91.9, $177.1)	$12.4 ($5.1, $21.0)	$40.4 ($26.9, $57.5)	$236.7 ($185.6, $291.4)
**Connecticut**	$13.1 ($8.0, $18.3)	$19.9 ($14.8, $25.8)	$6.5 ($4.5, $9.0)	$2.1 ($0.7, $3.9)	$0.9 ($0.1, $1.8)	$98.2 ($70.5, $131.8)	$8.7 ($3.6, $14.6)	$27.6 ($19.0, $38.8)	$177.0 ($142.0, $215.7)
**Delaware**	$4.1 ($2.6, $5.6)	$5.8 ($4.3, $7.5)	$1.8 ($1.2, $2.5)	$0.6 ($0.2, $1.1)	$0.2 ($0.0, $0.4)	$28.7 ($21.1, $38.0)	$2.1 ($0.9, $3.4)	$7.4 ($5.2, $10.2)	$50.8 ($41.1, $61.7)
**District of Columbia**	$3.9 ($2.3, $5.6)	$4.8 ($3.2, $6.5)	$1.0 ($0.6, $1.4)	$0.3 ($0.1, $0.8)	$0.1 ($0.0, $0.1)	$26.5 ($19.1, $35.1)	$1.4 ($0.5, $2.4)	$6.4 ($4.5, $8.6)	$44.3 ($35.3, $53.7)
**Florida**	$84.7 ($53.6, $116.1)	$125.9 ($93.5, $162.8)	$41.2 ($28.6, $57.3)	$14.5 ($5.1, $27.1)	$6.1 ($0.7, $11.3)	$591.8 ($430.5, $785.4)	$45.8 ($19.1, $75.6)	$151.6 ($104.7, $211.9)	$1,061.7 ($859.1, $1,287.8)
**Georgia**	$43.4 ($27.2, $60.6)	$61.4 ($44.3, $80.3)	$15.8 ($10.8, $21.8)	$5.2 ($2.1, $9.5)	$1.9 ($0.5, $3.4)	$317.3 ($230.7, $417.8)	$21.9 ($9.3, $36.1)	$82.8 ($58.2, $113.4)	$549.7 ($444.9, $665.6)
**Hawaii**	$4.3 ($2.3, $6.9)	$7.9 ($5.1, $11.4)	$2.3 ($1.4, $3.4)	$1.5 ($0.1, $4.2)	$0.4 ($0.1, $0.8)	$46.8 ($32.5, $63.9)	$4.3 ($1.5, $7.4)	$17.2 ($9.7, $26.7)	$84.8 ($66.6, $103.9)
**Idaho**	$4.8 ($2.6, $7.0)	$7.4 ($5.2, $9.8)	$2.7 ($1.8, $3.8)	$0.9 ($0.2, $1.6)	$0.4 ($0.0, $0.7)	$36.8 ($25.4, $50.4)	$3.6 ($1.3, $6.1)	$11.2 ($7.3, $16.1)	$67.7 ($52.6, $84.0)
**Illinois**	$47.7 ($29.5, $66.6)	$69.3 ($51.8, $88.8)	$21.0 ($14.5, $29.0)	$7.0 ($2.7, $12.7)	$2.9 ($0.5, $5.4)	$354.4 ($256.8, $471.0)	$29.3 ($12.5, $48.1)	$98.9 ($68.8, $138.3)	$630.5 ($509.1, $768.1)
**Indiana**	$22.6 ($13.4, $31.8)	$33.8 ($25.0, $44.4)	$11.0 ($7.6, $15.3)	$3.5 ($1.2, $6.4)	$1.6 ($0.1, $3.0)	$168.8 ($120.7, $227.7)	$15.0 ($5.9, $25.3)	$47.9 ($32.3, $68.5)	$304.1 ($242.0, $373.6)
**Iowa**	$10.0 ($5.5, $14.3)	$15.5 ($11.0, $20.5)	$5.5 ($3.7, $7.8)	$1.8 ($0.5, $3.3)	$0.8 ($0.0, $1.6)	$74.8 ($52.3, $101.8)	$7.2 ($2.6, $12.3)	$21.7 ($14.3, $31.1)	$137.2 ($107.8, $169.4)
**Kansas**	$9.4 ($5.4, $13.3)	$14.3 ($10.5, $18.8)	$4.8 ($3.3, $6.7)	$1.5 ($0.5, $2.8)	$0.7 ($0.1, $1.3)	$71.4 ($50.9, $96.4)	$6.6 ($2.7, $11.1)	$21.0 ($14.1, $30.0)	$129.6 ($102.5, $159.1)
**Kentucky**	$15.3 ($9.0, $21.5)	$23.0 ($16.9, $30.3)	$7.6 ($5.2, $10.7)	$2.4 ($0.8, $4.5)	$1.1 ($0.1, $2.1)	$113.8 ($81.3, $154.0)	$10.2 ($3.8, $17.3)	$32.0 ($21.4, $46.1)	$205.4 ($163.0, $252.5)
**Louisiana**	$21.3 ($13.6, $29.6)	$29.6 ($21.5, $38.7)	$7.8 ($5.3, $10.7)	$2.6 ($1.1, $4.8)	$0.9 ($0.2, $1.7)	$150.8 ($109.6, $199.4)	$10.1 ($4.2, $16.7)	$37.9 ($26.5, $51.9)	$261.0 ($212.2, $316.2)
**Maine**	$4.6 ($2.6, $6.7)	$7.2 ($5.1, $9.6)	$2.8 ($1.9, $3.9)	$0.9 ($0.2, $1.7)	$0.4 ($0.0, $0.8)	$33.9 ($23.5, $46.2)	$3.3 ($1.1, $5.7)	$9.5 ($6.2, $13.8)	$62.5 ($48.9, $77.9)
**Maryland**	$27.4 ($17.5, $37.7)	$37.9 ($27.6, $49.3)	$10.1 ($6.9, $13.9)	$3.5 ($1.4, $6.4)	$1.2 ($0.3, $2.2)	$196.3 ($143.3, $257.3)	$13.4 ($5.8, $21.8)	$50.5 ($35.6, $68.8)	$340.4 ($278.0, $409.7)
**Massachusetts**	$23.4 ($14.0, $32.7)	$35.7 ($26.4, $46.7)	$11.7 ($8.1, $16.2)	$3.8 ($1.3, $6.8)	$1.8 ($0.2, $3.3)	$177.9 ($127.5, $238.5)	$16.6 ($7.0, $27.8)	$51.8 ($35.3, $73.2)	$322.6 ($258.2, $395.2)
**Michigan**	$38.3 ($24.1, $53.0)	$56.1 ($41.9, $72.2)	$18.0 ($12.4, $25.0)	$5.9 ($2.1, $10.9)	$2.5 ($0.3, $4.6)	$278.3 ($201.3, $372.8)	$23.0 ($9.3, $38.2)	$75.4 ($51.8, $106.2)	$497.5 ($400.8, $606.7)
**Minnesota**	$17.1 ($9.7, $24.5)	$26.8 ($19.4, $35.4)	$9.2 ($6.3, $12.9)	$2.9 ($0.9, $5.3)	$1.3 ($0.1, $2.5)	$134.2 ($94.9, $181.1)	$13.0 ($5.4, $21.9)	$40.8 ($27.6, $58.1)	$245.3 ($194.4, $302.0)
**Mississippi**	$14.6 ($9.2, $20.3)	$20.0 ($14.4, $26.4)	$5.2 ($3.5, $7.2)	$1.8 ($0.7, $3.3)	$0.6 ($0.1, $1.2)	$100.5 ($73.0, $132.8)	$6.3 ($2.6, $10.5)	$24.4 ($17.0, $33.3)	$173.3 ($140.2, $210.8)
**Missouri**	$22.3 ($13.5, $30.9)	$33.1 ($24.6, $43.1)	$10.7 ($7.5, $15.0)	$3.5 ($1.2, $6.5)	$1.5 ($0.1, $2.9)	$163.2 ($117.4, $219.7)	$13.8 ($5.6, $23.2)	$44.9 ($30.5, $63.6)	$293.1 ($235.1, $358.4)
**Montana**	$3.3 ($1.8, $4.7)	$5.1 ($3.6, $6.8)	$2.0 ($1.3, $2.8)	$0.6 ($0.2, $1.2)	$0.3 ($0.0, $0.5)	$25.0 ($17.4, $34.0)	$2.4 ($0.9, $4.1)	$7.5 ($5.0, $10.7)	$46.3 ($36.3, $57.1)
**Nebraska**	$5.9 ($3.3, $8.5)	$9.1 ($6.5, $12.0)	$3.1 ($2.1, $4.4)	$1.0 ($0.3, $1.8)	$0.5 ($0.0, $0.9)	$45.0 ($31.6, $61.1)	$4.2 ($1.6, $7.2)	$13.4 ($9.0, $19.1)	$82.2 ($64.7, $101.0)
**Nevada**	$9.5 ($5.9, $13.2)	$14.6 ($10.8, $18.8)	$4.7 ($3.3, $6.5)	$1.7 ($0.6, $3.1)	$0.6 ($0.2, $1.1)	$75.7 ($55.1, $100.5)	$6.5 ($2.9, $10.5)	$22.3 ($15.5, $30.9)	$135.6 ($109.8, $164.5)
**New Hampshire**	$4.3 ($2.4, $6.2)	$6.7 ($4.8, $9.0)	$2.5 ($1.7, $3.5)	$0.8 ($0.2, $1.5)	$0.4 ($0.0, $0.7)	$33.1 ($23.0, $45.2)	$3.3 ($1.2, $5.8)	$9.7 ($6.3, $14.1)	$60.9 ($47.3, $76.0)
**New Jersey**	$33.6 ($21.4, $46.4)	$49.6 ($37.1, $63.5)	$15.2 ($10.6, $20.8)	$5.2 ($1.9, $9.4)	$2.2 ($0.5, $4.0)	$251.2 ($184.6, $330.8)	$21.3 ($9.5, $34.5)	$70.5 ($49.5, $97.9)	$448.7 ($364.7, $542.6)
**New York**	$78.4 ($49.4, $108.7)	$113.4 ($84.7, $144.6)	$33.4 ($23.4, $45.7)	$11.7 ($4.6, $20.8)	$4.7 ($1.1, $8.6)	$579.9 ($426.9, $762.3)	$46.2 ($20.8, $75.4)	$158.2 ($111.8, $219.9)	$1,025.8 ($833.4, $1,238.8)
**New Mexico**	$6.5 ($3.7, $9.1)	$10.1 ($7.3, $13.2)	$3.6 ($2.5, $5.0)	$1.2 ($0.4, $2.3)	$0.5 ($0.1, $1.0)	$50.2 ($35.6, $67.3)	$4.8 ($2.0, $7.9)	$15.6 ($10.6, $22.1)	$92.5 ($73.2, $112.8)
**North Carolina**	$42.0 ($26.6, $58.1)	$59.2 ($43.8, $76.2)	$17.2 ($11.9, $23.7)	$5.8 ($2.3, $10.5)	$2.3 ($0.4, $4.2)	$298.0 ($218.2, $395.7)	$22.2 ($9.7, $36.0)	$79.0 ($55.1, $109.5)	$525.5 ($426.1, $638.2)
**North Dakota**	$2.2 ($1.2, $3.2)	$3.5 ($2.5, $4.6)	$1.2 ($0.8, $1.7)	$0.4 ($0.1, $0.7)	$0.2 ($0.0, $0.3)	$17.4 ($12.1, $23.6)	$1.7 ($0.7, $2.9)	$5.5 ($3.7, $7.9)	$32.0 ($25.0, $39.5)
**Ohio**	$43.8 ($26.9, $60.3)	$64.4 ($47.9, $83.5)	$20.8 ($14.4, $28.9)	$6.7 ($2.4, $12.5)	$2.9 ($0.3, $5.6)	$317.2 ($228.4, $426.0)	$26.7 ($10.6, $44.6)	$86.0 ($58.6, $122.2)	$568.4 ($456.1, $695.4)
**Oklahoma**	$12.9 ($7.9, $17.9)	$19.8 ($14.7, $25.5)	$6.4 ($4.5, $9.0)	$2.3 ($0.8, $4.1)	$1.0 ($0.2, $1.8)	$100.1 ($72.7, $133.1)	$9.2 ($4.2, $14.8)	$31.4 ($21.5, $43.8)	$183.0 ($147.5, $221.7)
**Oregon**	$12.9 ($7.2, $18.4)	$20.0 ($14.3, $26.4)	$7.2 ($4.9, $10.2)	$2.4 ($0.7, $4.4)	$1.1 ($0.1, $2.0)	$98.5 ($69.2, $133.6)	$9.5 ($3.8, $16.0)	$29.5 ($19.8, $41.6)	$181.0 ($142.3, $222.7)
**Pennsylvania**	$48.7 ($29.7, $67.0)	$72.9 ($54.2, $94.8)	$24.1 ($16.7, $33.5)	$7.9 ($2.7, $14.7)	$3.6 ($0.3, $6.7)	$352.9 ($254.4, $474.6)	$30.2 ($12.1, $50.3)	$95.8 ($65.4, $135.6)	$636.0 ($512.4, $777.2)
**Rhode Island**	$3.7 ($2.2, $5.2)	$5.7 ($4.2, $7.5)	$1.9 ($1.3, $2.6)	$0.6 ($0.2, $1.1)	$0.3 ($0.0, $0.6)	$27.9 ($19.9, $37.6)	$2.6 ($1.1, $4.4)	$8.1 ($5.5, $11.5)	$50.8 ($40.5, $62.3)
**South Carolina**	$22.6 ($14.4, $30.9)	$31.2 ($22.9, $40.5)	$8.9 ($6.1, $12.3)	$3.1 ($1.2, $5.6)	$1.1 ($0.2, $2.1)	$155.3 ($113.1, $205.7)	$10.5 ($4.5, $17.1)	$38.6 ($26.9, $52.9)	$271.4 ($221.2, $328.4)
**South Dakota**	$2.6 ($1.4, $3.7)	$4.1 ($2.9, $5.4)	$1.5 ($1.0, $2.1)	$0.5 ($0.1, $0.9)	$0.2 ($0.0, $0.4)	$19.9 ($14.0, $27.0)	$2.0 ($0.8, $3.3)	$6.4 ($4.3, $9.1)	$37.1 ($29.2, $45.5)
**Tennessee**	$25.8 ($16.1, $35.9)	$37.5 ($27.8, $48.3)	$11.6 ($8.0, $16.0)	$3.8 ($1.4, $7.0)	$1.6 ($0.2, $3.0)	$185.8 ($134.7, $247.6)	$14.8 ($6.1, $24.7)	$49.8 ($34.1, $70.1)	$330.6 ($266.2, $403.8)
**Texas**	$87.4 ($53.4, $122.9)	$129.2 ($96.1, $167.2)	$37.8 ($26.3, $52.7)	$12.2 ($4.3, $22.2)	$5.1 ($0.9, $9.4)	$680.7 ($491.1, $912.3)	$58.6 ($24.6, $97.5)	$201.0 ($138.4, $283.0)	$1,212.2 ($975.9, $1,480.5)
**Utah**	$7.3 ($3.7, $11.0)	$11.0 ($7.7, $14.6)	$3.5 ($2.4, $5.0)	$1.1 ($0.3, $2.0)	$0.5 ($0.0, $0.9)	$59.7 ($40.8, $82.4)	$5.9 ($2.2, $10.3)	$20.0 ($13.1, $29.1)	$108.9 ($84.1, $136.0)
**Vermont**	$2.1 ($1.2, $3.0)	$3.3 ($2.3, $4.4)	$1.2 ($0.8, $1.7)	$0.4 ($0.1, $0.7)	$0.2 ($0.0, $0.3)	$15.8 ($10.9, $21.5)	$1.6 ($0.5, $2.7)	$4.5 ($2.9, $6.6)	$29.0 ($22.6, $36.1)
**Virginia**	$33.4 ($20.9, $46.1)	$47.8 ($35.6, $61.2)	$13.7 ($9.6, $19.0)	$4.7 ($1.8, $8.3)	$1.8 ($0.4, $3.3)	$246.7 ($180.6, $325.7)	$19.2 ($8.5, $31.4)	$67.7 ($47.7, $93.8)	$435.0 ($352.7, $527.8)
**Washington**	$22.0 ($12.7, $31.1)	$34.4 ($25.1, $44.8)	$11.6 ($8.0, $16.2)	$3.9 ($1.2, $7.1)	$1.7 ($0.3, $3.1)	$177.0 ($125.9, $237.1)	$17.1 ($7.5, $27.9)	$55.4 ($37.8, $77.6)	$323.0 ($256.8, $393.7)
**West Virginia**	$6.6 ($3.7, $9.3)	$10.1 ($7.2, $13.4)	$3.7 ($2.5, $5.2)	$1.2 ($0.4, $2.2)	$0.5 ($0.0, $1.1)	$47.8 ($33.6, $65.0)	$4.4 ($1.6, $7.5)	$13.0 ($8.6, $18.8)	$87.3 ($68.9, $107.9)
**Wisconsin**	$19.2 ($11.1, $27.2)	$29.5 ($21.4, $38.9)	$10.2 ($7.0, $14.4)	$3.2 ($1.0, $6.0)	$1.5 ($0.1, $2.8)	$145.4 ($102.8, $196.4)	$13.6 ($5.3, $23.2)	$42.3 ($28.4, $60.7)	$265.0 ($210.0, $326.1)
**Wyoming**	$1.8 ($1.0, $2.6)	$2.7 ($1.9, $3.6)	$1.0 ($0.7, $1.4)	$0.3 ($0.1, $0.6)	$0.1 ($0.0, $0.3)	$13.9 ($9.6, $18.9)	$1.3 ($0.5, $2.3)	$4.2 ($2.7, $6.0)	$25.3 ($19.6, $31.4)
**National^1^**	$1,165 ($712, $1,626) ($187.2, $2,213)	$1,724 ($1,271, $2,238) ($274.6, $1724.4)	$536 ($371, $745) ($90.4, $1,076.2)	$182 ($64, $335) ($23.1, $423.1)	$76 ($12, $140) ($4.7, $191.3)	$8,721 ($6,300, $11,625) ($172.4, $16,813.4)	$733 ($309, $1,213) ($92.8, $1,595.7)	$2,460 ($1,690, $3,450) ($424.9, $4830.3)	$15,599 ($12,544, $18,992) ($2,684.5, $29,521.5)

^1^ All confidence intervals with the exception of the second set in national totals are based on 1000 Monte Carlo simulations holding the global parameter (increased disease cost of malnutrition) constant. This allows states to be compared to each other and to the national total. The second set of confidence intervals for the national totals are based on 1000 Monte Carlo simulations where the global parameter varies according to the distribution outlined in S1 Appendix and are included to describe the sensitivity of the overall results.

We estimate that the burden of DAM for the 8 diseases studied was over $15.5 billion or $48 per capita annually. This is greater than the most recent comparable estimate from Snider et al. which estimated the direct medical cost of DAM for these same diseases to be $10.4 billion (adjusted for inflation) [16]. There are two primary reasons for this gap. First, Snider et al.’s estimate was based on 2 years of NHANES data (2009–2010) while our findings reflect 6 years (2009–2014), which results in different within disease estimates of malnutrition. Second, we attempted to use the most current literature for our estimates of disease costs, to reflect changes in the health care landscape. Therefore our costs were different from Snider et al.’s for some specific diseases. Our per capita cost is higher than Snider et al.’s ($48 vs. $32), much closer to the European per capita DAM medical cost estimate of $45.

**Fig 2:**
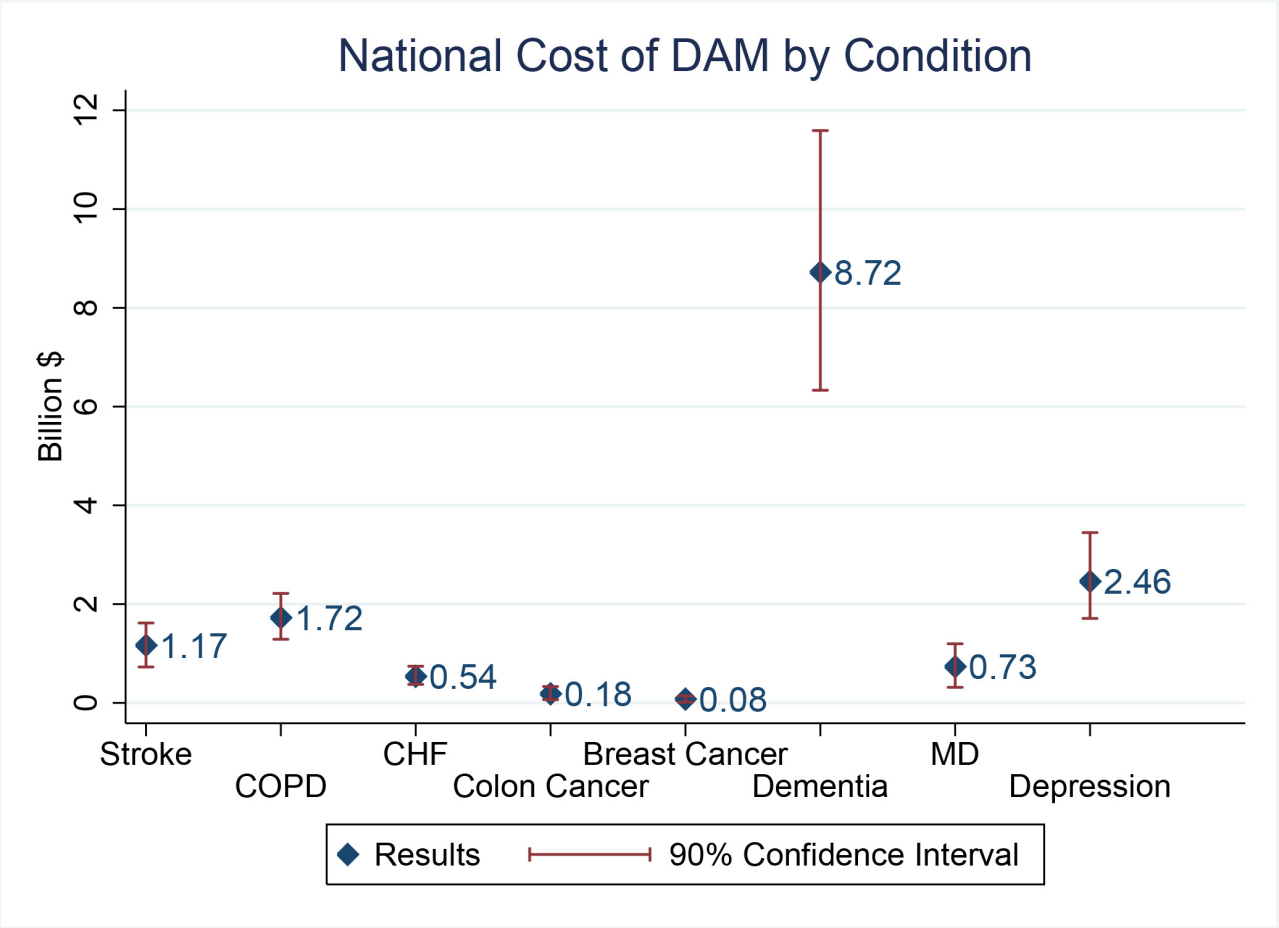
National Cost of DAM by condition.

Comparison of the contributions of different diseases to the cost of DAM reveals that dementia is by far the greatest contributor, costing the United States over $8.7 billion annually (Fig 2). This is primarily because the average annual medical spending on dementia patients is high ($36,397) [27], the prevalence of dementia is high (7% using NHANES data) and malnutrition among patients with dementia is high (7% using NHANES data). The next closest driver of cost is depression, which costs $2.46 billion annually.

Because the age 65 and over population is of particular interest to policy makers and healthcare providers, we also report results for this age group. Although only 14% of the population are age 65 and older, they account for 28% ($4.3 billion) of the total U.S. burden of DAM, and the per capita cost for the elderly is almost double that of the general population ($93 per capita for aged 65+ vs. $48 per capita for the general population). This cost reflects the higher disease prevalence among older adults. For example, the prevalence of dementia in those age 65 and over is higher than the general population (12.5% vs. 6%, p<0.01), and older adult dementia patients are no less likely to be malnourished (6.0% older adult dementia patients vs. 7.8% general population dementia patients, p = 0.60)

## Discussion

The field of health economics and nutrition is an emerging area of research. To our knowledge this is the first study to explore the economic burden of DAM at the state level. Increasing healthcare costs are a major concern, particularly in our aging society. It is likely the progression of DAM can be mitigated through optimizing nutritional care. The size of the economic burden of DAM nationally, at the state level and on a per capita basis indicates a need for systematic research in this area.

Most changes in the healthcare system occur at the local and state levels. The data from this study provides evidence for state policy makers and hospital administrators to develop action plans and policy changes (such as instituting a malnutrition quality measure in hospitals) which promote change in clinical practices and health outcomes that ultimately will decrease healthcare costs. Malnutrition is costly to our healthcare system and proper nutrition can lessen this cost. One area where reduction of DAM holds promise for dramatic cost reductions is hospital readmissions. Federal healthcare reform and implementation of the Readmission Reduction Program penalize hospitals for high readmission rates. Nutritional interventions have been shown to reduce readmission rates [28,29]. Consumption of oral nutrition supplements in hospitalized patients significantly decreased the probability for 30 day readmission, length of stay, and health care cost [28]. Meehan et al. found treating patients at risk for malnutrition with oral nutrition supplementation reduced incidences of pressure ulcers, length of stay, 30 day readmissions and costs of care [30]. Identification of malnutrition or risk for malnutrition in the hospital and prior to discharge provides an opportunity to tailor home-based nutritional interventions after discharge. A nutrition assessment just prior to discharge with a nutrition care plan for patients with or at risk for developing malnutrition seems warranted.

Malnutrition is also a concern for transitions of care. The lack of standardized malnutrition screening means that there is not a consistent link to connect malnutrition care between hospitals, nursing homes, home, and community settings. Watson et al. [31] recommended approaching the issue in a “multisystem, not just multidisciplinary way, as policy makers, health systems, and healthcare professionals all play roles.” An ICD-10 code for sarcopenia will be available in October 2016. It is hoped this will provide a basis for the development of a broader universally accepted definition for DAM.

Numerous studies have reported patients who remained well-nourished during hospitalization had lower health care costs compared to those who became malnourished [12,32–34]. Additionally, several recent studies have explored the cost effectiveness of providing nutrition supplementation to at-risk [35–39]. The NOURISH Study Group is the largest randomized controlled clinical trial (N = 652) investigation to date of the effectiveness of oral nutrition supplements, oral ingestion of supplementary foods for medical reasons. They found older, malnourished patients randomized to high-protein oral nutritional supplement for 90 days had improved nutritional status and decreased mortality compared to those randomized to a placebo [40].

There is evidence that proper nutrition can help improve clinical outcomes for malnourished patients with specific chronic diseases. Reduced food intake and altered metabolism in cancer patients puts them at risk for weight loss and is associated with unique complications including decreased response to therapy and increased toxicity of chemotherapy (often requiring decreased doses, limiting effectiveness) [41,42]. Clinical trials have shown cancer patients provided nutrition therapy supplemented with nutrients supporting immune function to have reduced risks of complications, decreased length of antibiotic therapy, and shortened length of stay [43–45]. A recent review completed by a Task Force formed by the European Respiratory Society determined proper nutrition in COPD patients can have pulmonary, metabolic, and cardiovascular risk benefits [46]. Compared to typical therapy, nutritional therapy combined with exercise reduced hospital costs in muscle-wasted COPD patients [47]. Older patients with acute ischemic stroke provided an enteral formula including whey protein had better clinical outcomes compared to patients provided the same formula with protein coming from casein [48].

Our study is a model, and thus reflects estimates based on public data versus actual costs calculated from individual patient charges. Some of the limitations of our study include the limited number of diseases considered, medical cost estimates from the literature (as opposed to primary data), the limited variation in the marginal cost of malnutrition by disease state, and the lack of a universally accepted definition of malnutrition. Additionally, our model estimates aggregate direct medical costs borne by society, but is agnostic about how those costs are distributed between payers, consumer and government.

## Conclusions

The findings are important to state policy makers and those involved in healthcare decision-making roles focused on reducing healthcare costs. The joint area of clinical nutrition support and health economics is emerging, and is needed for value-based healthcare decisions. Under healthcare payment reform, healthcare providers are held accountable for both costs and quality [49]. Our study is one of the first to quantify the state-level burden of DAM. It comes at a critical time when continued implementation of U.S. healthcare reform provides an opportunity to bring increased awareness to malnutrition-related issues in the healthcare system so they can be addressed and help improve patient health outcomes and lower healthcare costs.

## Supporting Information

S1 Appendix Table A: Model Parameters. Table B: Disease Definitions (adapted from Snider et al. 2014).
(DOCX)
