# Supplementary material for: Economic Burden of Disease-Associated Malnutrition at the State Level
Source: PLoS One. 2016 Sep 21;11(9):e0161833. doi: 10.1371/journal.pone.0161833 (PMC5031313; doi:10.1371/journal.pone.0161833)
Supplement: S1 Appendix — Table A: Model Parameters. Table B: Disease Definitions (adapted from Snider et al. 2014). (DOCX) [file pone.0161833.s001.docx]

**Appendix**

Table A: Model Parameters

| Parameter | Value | Monte Carlo Distribution |
| --- | --- | --- |
| Annual Direct Medical Cost |  |  |
| Stroke([1](#_ENREF_1)) | $14,260 | Uniform($11408, $17112) |
| Breast Cancer([2](#_ENREF_2)) | $5,206 | Uniform($4164, $6247) |
| Colorectal Cancer([2](#_ENREF_2)) | $12,698 | Uniform ($10158, $15238) |
| Coronary Heart Disease([3](#_ENREF_3)) | $5,266 | Uniform ($4213, $6319) |
| COPD([4](#_ENREF_4)) | $10,164 | Uniform ($8131, $12197) |
| Dementia([5](#_ENREF_5)) | $36,398 | Gamma (51.5, 705.8)* |
| Musculoskeletal Disorders([6](#_ENREF_6)) | $8,223 | Uniform ($6578, $9868) |
| Depression([7](#_ENREF_7)) | $10,765 | Gamma (54.2, $198.7)* |
| Global Parameter |  |  |
| Increased Cost of Malnutrition([8](#_ENREF_8)) | 1.193 | Normal (1.193, 0.09)* |

*Parameters modeled using distribution data from publication

Table B: Disease Definitions (adapted from Snider et al. 2014):

| Disease State | NHANES Definition  (Malnutrition within Disease) | NHIS Definition  (Disease Prevalence) |
| --- | --- | --- |
| Stroke | Answered yes to “Ever told you had a stroke?” (MCQ160F) | NHIS adult sample: Have you EVER been told by a doctor or other health professional that you had ...A stroke? (STREV) OR Stroke problem causes difficulty with activity (aflhca8) NHIS child sample: Not used. NHANES used instead. |
| COPD | Answered yes to either of these questions:   - Ever told you had chronic bronchitis? (MCQ160K) or - Ever told you had emphysema (MCQ160G) | NHIS adult sample: DURING THE PAST 12 MONTHS, have you been told by a doctor or other health professional that you had...Chronic bronchitis? (CBRCHYR); OR Have you EVER been told by a doctor or other health professional that you had...Emphysema?(EPHEV); OR Lung/breathing problem (e.g., asthma and emphysema) causes difficulty with activity (AFLHCA11) NHIS child sample: Not used. NHANES used instead. |
| Coronary Heart Disease | Answered yes to ANY of these questions:   - Ever told you had congestive heart failure (MCQ160B) - Ever told you had coronary heart disease (MCQ160C) - Ever told you had angina/angina pectoris (MCQ160D) | NHIS adult sample: Have you EVER been told by a doctor or other health professional that you had... Coronary heart disease (CHDEV)? OR ...A heart attack (also called myocardial infarction) (MIEV)? OR ... Angina, also called angina pectoris (ANGEV)? NHIS child sample: Not used. NHANES used instead. |
| Depression | Score of 10 or higher on the Patient Health Questionnaire (PHQ-9) | NHANES Used |
| Colorectal Cancer | Responded Colon OR Rectum to which type of cancer (MCQ230A, MCQ230B, MCQ230C, MCQ230D) | NHIS adult sample: Have you EVER been told by a doctor or other health professional that you had...Cancer or a malignancy of any kind? What kind of cancer was it? (CNKIND7 CNKIND21) (Colon or Rectum) NHIS child sample: Not used. NHANES used instead. |
| Breast Cancer | Responded “Breast” to which type of cancer (MCQ230A, MCQ230B, MCQ230C, MCQ230D) | NHIS adult sample: Have you EVER been told by a doctor or other health professional that you had...Cancer or a malignancy of any kind? (CANEV) What kind of cancer was it? (CNKIND5)(Breast) NHIS child sample: Not used. NHANES used instead. |
| Dementia | Respondent indicated yes to:   - Are you limited in any way because of difficulty remembering or because you experienced periods of confusion (PFQ057) OR   Responded “Senility” was a health problem that caused difficulty (PFQ063B, PFQ063C, PFQ063D, PFQ063E) | NHANES Used |
| Musculo-skeltal Disorders | Positive response to:   - Ever told you had arthritis (MCQ160A) OR   answered arthritis/rheumatism to question: What health problems causing difficulty? (PFQ063A, PFQ063B, PFQ063C, PFQ063D, PFQ063E) Or  Answered yes to any of the following:   - Had neck/back/hip pain for 3 or more months in a row? (ARQ024A, ARQ024B, ARQ024C, ARQ024D, ARQ024E, ARQ024F, ARQ024G) - Was the cause of the pain gout? (ARQ118AJ) Ever told you had Iritis/Uveitis (ARD125A) Ever told you had Ankylosing Spondylitis (ARQ125E) Ever told you had osteoporosis/brittle bones (OSQ060) OR   Recorded these measurements:   - Occupit to Wall Distance > 2 cm (ARXO2WD) - Lumbar flexion < 5 cm (ARDLFTL) - AND - Takes any of the following medications: - riluzole, betaseron, avonex, rebif, glatiramer, tocilizumab, anakinra, kineret OR - Takes inocin or indomethacin for pain? (ARQ030C) o Takes cox-2 inhibitor (Celebrex, Vioxx) for pain (ARQ030D) | NHIS adult sample: Not used. NHANES used instead. NHIS child sample: Ever told SC had arthritis (CCONDL08) |

**References**

1. Thomas N. Taylor PHD, James C. Torner, Julia Holmes, Jay W. Meyer, and Mark F. Jacobson. Lifetime Cost of Stroke in the United States. Stroke. 1996;27:1459-66.

2. Mariotto AB, Robin Yabroff K, Shao Y, Feuer EJ, Brown ML. Projections of the Cost of Cancer Care in the United States: 2010–2020. JNCI Journal of the National Cancer Institute. 2011;103(2):117-28.

3. Russell MWM, Huse DMMA, Drowns SMA, Hamel ECAB, Hartz SCS. Direct Medical Costs of Coronary Artery Disease in the United States. Am J Cardiol.81(9):1110-5.

4. Ford ES, Murphy LB, Khavjou O, Giles WH, Holt JB, Croft JB. Total and State-Specific Medical and Absenteeism Costs of COPD Among Adults Aged 18 Years in the United States for 2010 and Projections Through 2020. Chest. 2015;147(1):31-45.

5. Hurd MD, Martorell P, Delavande A, Mullen KJ, Langa KM. Monetary Costs of Dementia in the United States. N Engl J Med. 2013;368(14):1326-34.

6. The Burden of Musculoskeletal Diseases in the United States (BMUS), Third Edition2014. Available from: <http://www.boneandjointburden.org/2014-report/xd0/musculoskeletal-medical-care-expenditures>.

7. Greenberg PE, Fournier A-A, Sisitsky T, Pike CT, Kessler RC. The economic burden of adults with major depressive disorder in the United States (2005 and 2010). The Journal of clinical psychiatry. 2015;76(2):1,478-162.

8. Amaral TF, Matos LC, Tavares MM, Subtil A, Martins R, Nazare M, et al. The economic impact of disease-related malnutrition at hospital admission. Clin Nutr. 2007;26(6):778-84.
